# First Report of *Colletotrichum fructicola*, *C. rhizophorae* sp. nov. and *C. thailandica* sp. nov. on Mangrove in Thailand

**DOI:** 10.3390/pathogens12121436

**Published:** 2023-12-10

**Authors:** Chada Norphanphoun, Kevin D. Hyde

**Affiliations:** 1 Center of Excellence in Fungal Research, Mae Fah Luang University, Chiang Rai 57100, Thailand; oomchn@gmail.com; 2 School of Science, Mae Fah Luang University, Chiang Rai 57100, Thailand; 3 Mushroom Research Foundation, 128 M.3 Ban Pa Deng T. Pa Pae, A. Mae Taeng, Chiang Mai 50150, Thailand

**Keywords:** Ascomycota, molecular phylogeny, phylogeny, taxonomy, two new species

## Abstract

*Colletotrichum*, a genus within the phylum Ascomycota (Fungi) and family Glomerellaceae are important plant pathogens globally. In this paper, we detail four *Colletotrichum* species found in mangrove ecosystems. Two new species, *Colletotrichum rhizophorae* and *C. thailandica*, and a new host record for *Colletotrichum fructicola* were identified in Thailand. *Colletotrichum tropicale* was collected from Taiwan’s mangroves and is a new record for *Rhizophora mucronata*. These identifications were established through a combination of molecular analysis and morphological characteristics. This expanded dataset for *Colletotrichum* enhances our understanding of the genetic diversity within this genus and its associations with mangrove ecosystems. The findings outlined herein provide data on our exploration of mangrove pathogens in Asia.

## 1. Introduction

Mangroves, the coastal ecosystems where land and sea merge, have been a subject of fascination for ecologists, conservationists, and nature enthusiasts for decades. Thailand has an extensive coastline and is home to numerous mangrove forests that have not only drawn the attention of researchers but have also unveiled a lesser-known yet incredibly diverse facet of these ecosystems—the extraordinary diversity of fungi they harbor [1,2]. This introduction sets the stage for the exploration of the captivating world of Thai mangroves and their rich fungal diversity. Thailand’s mangrove forests are a critical component of its coastal biodiversity and ecological integrity. They serve as a protective buffer against erosion, tidal surges, provide invaluable breeding grounds for marine species, and contribute significantly to carbon sequestration and climate change mitigation [3]. While the spotlight is often on the charismatic fauna and flora within mangroves, the fungi inhabiting these environments have, until recently, remained less studied. Recent studies have shed light on the remarkable diversity of fungi in Thai mangroves [4,5,6,7]. These fungi exhibit unique adaptations to the harsh conditions of mangrove ecosystems, thriving in saline environments and forming intricate relationships with mangrove trees and other microorganisms [1,2]. They play pivotal roles in nutrient cycling, organic matter decomposition, and symbiotic associations, all of which are essential for the health and sustainability of mangrove ecosystems [8,9].

*Colletotrichum* is a genus within the phylum Ascomycota (Fungi), belonging to the order Glomerellales and family Glomerellaceae [10]. There are 1035 names in Index Fungorum (http://www.indexfungorum.org/, Access Date 18 September 2023) with the type species being *Colletotrichum lineola* Corda. The genus is characterized by hemibiotrophic or necrotrophic lifestyles, displaying a biotrophic phase during initial host colonization before transitioning to necrotrophic, leading to cell death [1,11]. *Colletotrichum* species are important plant pathogens causing anthracnose or *Colletotrichum* blight diseases, infecting a wide range of hosts, including fruits, vegetables, ornamental plants, and agricultural crops [12,13,14,15]. Typical symptoms of *Colletotrichum* infection include dark, sunken lesions with defined edges on leaves, stems, and fruits, resulting in wilting, rotting, and premature fruit drops [16,17]. Given its economic significance, *Colletotrichum* causes substantial losses in crop yield and quality globally, affecting major plants like mango, banana, *Citrus*, pepper, coffee, and strawberry [18,19,20,21]. The morphological features of *Colletotrichum* vary among species but generally include conidia, conidiomata, and setae [22]. Disease management involves using resistant cultivars, cultural practices, fungicides, and sanitation [23]. Molecular techniques, such as DNA sequencing of specific genes (e.g., ITS, *act*, *β-tubulin*, *gapdh*), evolutionary and coalescent-based methods, aid in accurate identification [24,25]. Ongoing research aims to understand pathogenic mechanisms, host specificity, and sustainable disease control strategies [26]. Some *Colletotrichum* species are also endophytes or latent pathogens, which means they live in plants without causing disease until the right conditions are met, including mangroves [27,28,29,30,31,32,33,34]. *Colletotrichum*’s prevalence and impact on mangroves have been thoroughly investigated in several studies, providing crucial data for a comprehensive understanding of its ecology and management strategies [28,35,36].

In this study, we studied the mangroves of Thailand and Taiwan to uncover the phylogenetic diversity of *Colletotrichum* species associated with *Rhizophora apiculata* and *R. mucronata*, respectively. The aim of this study was to identify these isolates based on phylogenetic data and morphology to confirm their novel associations in mangrove ecosystems.

## 2. Materials and Methods

### 2.1. Sampling and Examination of Specimens

Fresh leaf samples were collected in 2017 from *Rhizophora apiculata* in Thailand. Fresh specimens were taken to the laboratory in paper bags, examined, and described. Morphological characters of conidiomata were examined using an Olympus SZX16 stereo microscope (Olympus Corporation, Tokyo, Japan). Micromorphology was studied and photographed using a Nikon Eclipse Ni compound microscope with a Microscope Camera DS-Ri2 (Nikon Corporation, Tokyo, Japan). All image measurements were made with the Image Frame Work program v. 0.9.7 (Tarosoft ®, Nontha Buri, Thailand). Photoplates were made using Adobe Photoshop CC 2019 version 20.0.1 (Adobe Systems, San Jose, CA, USA).

The cultures were acquired using the tissue isolation technique as described in the study of Norphanphoun et al. [37]. Single hyphal tips were transferred onto 2% potato agar (PA) plates at room temperature (25 °C ± 2) throughout a one-week period: 12 hours dark and 12 hours light. The cultural features were observed and documented at intervals of 5, 7, and 14 days. The morphological characteristics of the culture were analyzed during the entire cultivation duration. In order to conduct further experiments, pure cultures were cultivated on potato dextrose agar (PDA) (HiMedia Laboratories LLC, Kennett Square, PA, USA). Dried and living cultures were deposited in the culture collection at Mae Fah Luang University (MFLUCC) and herbarium collection (MFLU), Chiang Rai, Thailand. The enumeration of Faces of Fungi (https://www.facesoffungi.org/) was conducted following the methodology outlined in Jayasiri et al. [38].

### 2.2. DNA Extraction, Amplification via PCR, and Sequencing

Genomic DNA was extracted from fresh fungal mycelia growing on PDA at room temperature (25 °C ± 2) for two weeks using an E.Z.N.A^®^ Fungal DNA Mini Kit, (Omega Bio-tek, Inc., Nocross, GA, USA) following the manufacturer’s protocols. Polymerase chain reactions (PCR) were carried out using the following primer pairs: ITS1/ITS4 to amplify the internal transcribed spacer region (ITS), ACT512F/ACT738R for actin (*act*), GDF1/GPDHR2 for partial glyceraldehyde-3-phosphate dehydrogenase region (*gapdh*), T1/T2 for beta-tubulin (*β-tubulin*), CHS-79F/CHS-354R for chitin synthase (*chs-1*), CL1C/CL2C for calmodulin (*cal*) [39,40,41,42].

The amplification reactions were carried out using the following protocol: 25 μL reaction volume containing 1 µL of DNA template, 1 µL (20 µM stock concentration) of each forward and reverse primers, 12.5 µL of DreamTaq Green PCR Master Mix (2×) (Thermo Fisher Scientific Inc., Waltham, MA, USA), and 9.5 µL of double-distilled water (ddH_2_O). The PCR thermal cycling program for each locus is described in Table 1. PCR products were analyzed using 1.7% TAE agarose gels containing the 100 bp DNA Ladder RTU (Bio-Helix Co., Ltd., Taipei, Taiwan) to confirm the presence of amplicons at the expected molecular weight. The purification and sequencing of PCR products using the amplification primers specified above were conducted at SolGent Co., Ltd., located in Daejeon, Republic of Korea.

### 2.3. Phylogenetic Analysis

The raw readings were processed and organized into contigs using Geneious Prime^®^ 2023.2.1 Java Version 11.0.18+10 (64-bit) software (Biomatters Inc., Boston, MA, USA). The newly generated sequences were utilized as queries to conduct a BLASTn search against the nonredundant (nr) database in GenBank (https://www.ncbi.nlm.nih.gov/; accessed on 1 September 2023). The retrieval of similar sequences was conducted, followed by the construction of numerous alignments. The GenBank taxonomy browser was utilized to verify all sequences classified as *Colletotrichum* in the database. BioEdit version 7.2.5 (Ibis Biosciences, Carlsbad, CA, USA) [43] was used to assign open reading frames of the protein coding sequences of *actin*, *gapdh*, *β-tubulin*, *chs-1*, and *cal* according to reference sequences in the GenBank database. The combined sequence data of all loci were used to perform maximum likelihood (ML) and Bayesian inference analysis (BI). The dataset consisted of 126 taxa of the *Colletotrichum gloeosporioides* species complex and two taxa from singleton species as outgroups, *C. arecacearum* strains MH0003 and MH0003-1. Outgroup sequences were selected based on preliminary analysis of the multigene phylogeny of the *Colletotrichum* species complex dataset. All taxa used for these analyses can be found in Table 2.

Sequences were aligned for each locus separately using the MAFFT v.7.110 online program (http://mafft.cbrc.jp/alignment/server/; accessed on 19 September 2023) [44]. TrimAl/readAl v1.2. program was used to trim ambiguously aligned positions [45]. The software BioEdit version 7.2.5 was utilized to make additional manual edits as needed [43]. The congruency of genes and their potential for combination were assessed using a partition homogeneity test (PHT) conducted using PAUP* 4.0b10 software [46]. The concatenated sequence alignments were acquired from MEGA version 7.0.14 and version 10.1.0, as reported by Kumar et al. [47] and Tamura et al. [48], respectively. Geneious Prime^®^ 2023.2.1 was used to convert file format to Nexus BI analyses.

The data were divided into the following categories: ITS, *act*-exon, *gapdh*-exon, *β-tubulin*-exon, *chs-1*-exon, *cal*-exon, *act*-intron, *gapdh*-intron, *β-tubulin*-intron, and *cal*-intron. The researchers utilized the software RAxML-HPC2 on XSEDE to conduct maximum likelihood (ML) analysis, which was implemented using the CIPRES Science Gateway web server (https://www.phylo.org/portal2/; accessed on 20 November 2023) [49]. A total of 1000 bootstrap repeats were conducted in a swift manner, employing the GTRGAMMA model to simulate nucleotide evolution. The researchers conducted a Bayesian inference analysis by utilizing the Markov Chain Monte Carlo (MCMC) algorithm, which was implemented on the CIPRES Science Gateway web server. Specifically, they used MrBayes on XSEDE, as described by Miller et al. [49]. The optimal nucleotide substitution model for each partition was individually calculated using MrModeltest version 2.2 (Boston, MA, USA), as shown in Table 3 [50]. The computation of posterior probability involved the execution of two independent runs, each consisting of four chains. These runs were initiated from a randomly generated tree topology. A total of 10 million generations were executed for the given dataset. The sampling of trees occurred at regular intervals of 100 generations. According to Ronquist et al. [51], a quarter of the trees were excluded as burn-in values, while the average standard deviation of split frequencies reached convergence below 0.01.

The phylogram was generated using FigTree v1.4.3 (http://tree.bio.ed.ac.uk/software/figtree/) [52], a software tool commonly used for visualizing phylogenetic trees. The final figure was created using Adobe Illustrator CC version 23.0.1 (64-bit) and Adobe Photoshop CC version 20.0.1 release, both products developed by Adobe Systems in California, USA. The newly produced sequences in this investigation were deposited in GenBank as indicated in Table 2. The completed alignments and trees were submitted to TreeBASE.

The Genealogical Concordance Phylogenetic Species Recognition (GCPSR) model with a pairwise homoplasy index (PHI) test was used to analyze the newly generated taxon and its most phylogenetically close neighbors [53]. The PHI test was performed in SplitsTree v. 4.14.6 [54,55] with a five-locus concatenated dataset (ITS, *act*, *gapdh*, *β*-*tubulin*, *chs-1*, and *cal*) to determine the recombination level among phylogenetically closely related species. A pairwise homoplasy index below a 0.05 threshold (Φw < 0.05) indicated the presence of significant recombination in the dataset. The relationship between closely related species was visualized by constructing a split graph.

## 3. Results

The results of the partition homogeneity test (PHT) for the phylogenetic tree were not significant (95% level), which suggests that the individual datasets can be combined. To assess tree topology and clade support, single-locus phylogenetic trees were also generated before the combined gene tree was conducted. In this research, we introduce two novel *Colletotrichum* species alongside two known species.

**Figure 1:**
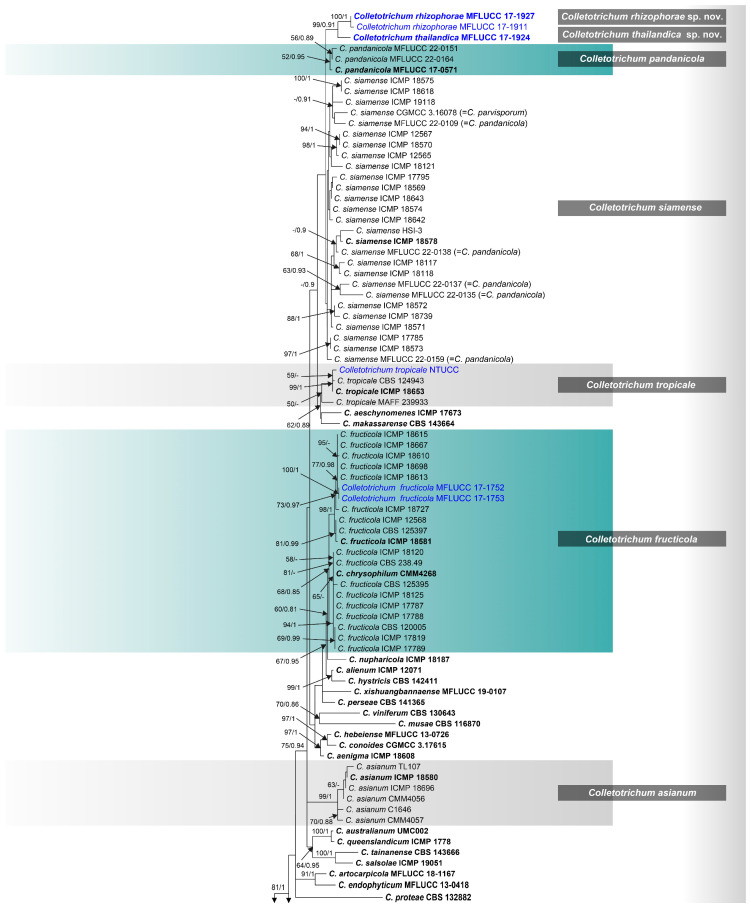

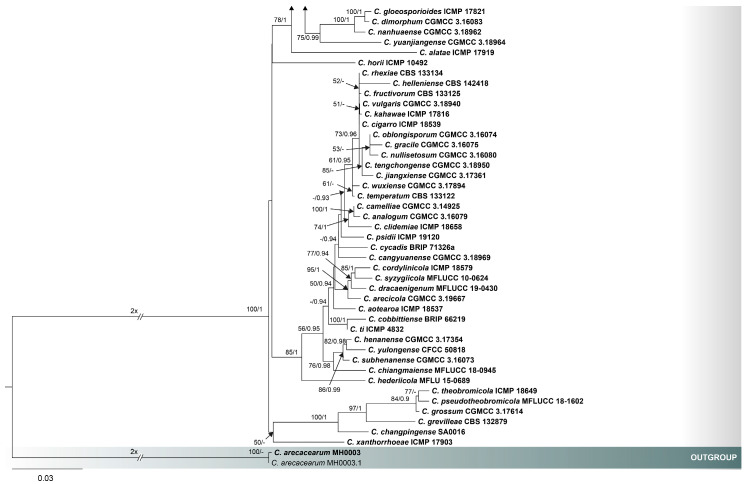
Phylogenetic tree generated from maximum likelihood analysis based on combined ITS, *act*-exon, *gapdh*-exon, *β-tubulin*-exon, *chs-1*-exon, *act*-intron, *gapdh*-intron, *β-tubulin*-intron sequence data. The species obtained in this study are in blue and species synonymized are in green. Ex-type taxa are in bold. Bar = 0.03, which represents the estimated number of nucleotide substitutions of site per branch.

The phylogenetic analysis utilized a comprehensive dataset encompassing six genes, including 126 strains from the *Colletotrichum* species in the gloeosporioides species complex and 2 singleton strains—*C. arecacearum* strains MH0003 and MH0003-1 sequences served as the outgroup. This dataset had a total length of 1803 characters, inclusive of alignment gaps, with the following partitions: ITS1+5.8S+ITS2 (1–593), *act*-exon (594–679), *gapdh*-exon (680–743), *β-tubulin*-exon (744–973), *chs-1*-exon (974–1197), *cal*-exon (1198–1569), *act*-intron (1570–1738), *gapdh*-intron (1739–1951), *β-tubulin*-intron (1952-2175), and *cal*-intron (2176–2534). Both maximum likelihood (ML) and Bayesian inference (BI) were employed for the analysis. Notably, trees generated under distinct optimality criteria exhibited congruent topologies and showed no significant differences. The highest-scoring likelihood tree for the combined dataset possessed a final likelihood value of -13,417.186937 (Figure 1). Within this tree, the new strains clustered within the gloeosporioides species complex clade, alongside other sequences identified as members of the gloeosporioides species complex. Remarkably, this species complex received robust statistical support, with 100% bootstrap support (BSML) and a posterior probability of 1.00 (PPBI).

The analysis of six genetic loci using both maximum likelihood (ML) and Bayesian inference (BI) methods resulted in a phylogenetic tree with well-supported clades, as shown in Figure 1. Within this study, we propose the recognition of two novel species, namely *C. rhizophorae* and *C. thailandica*, with robust statistical backing, signified by a high bootstrap support of 95% (BSML) and a posterior probability of 0.85 (PPBI). In terms of known species, two strains originating from mangrove habitats in Thailand (MFLUCC 17-1752 and MFLUCC 17-1753) were classified as members of the species *C. fructicola*, while a strain from Taiwan (NTUCC) was identified as *C. tropicale*. Notably, MFLUCC 17-1752 and MFLUCC 17-1753 clustered within the *C. fructicola* species group with substantial support: a 98% bootstrap support (BSML) and a posterior probability of 1.00 (PPBI). On the other hand, strain NTUCC was grouped within the *C. tropicale* species cluster, exhibiting a strong 99% bootstrap support (BSML) and a posterior probability of 1.00 (PPBI). It is noteworthy that all newly introduced strains in this study shared the same topological arrangement as the preliminary analysis of the *Colletotrichum* species complex.

**Figure 2:**
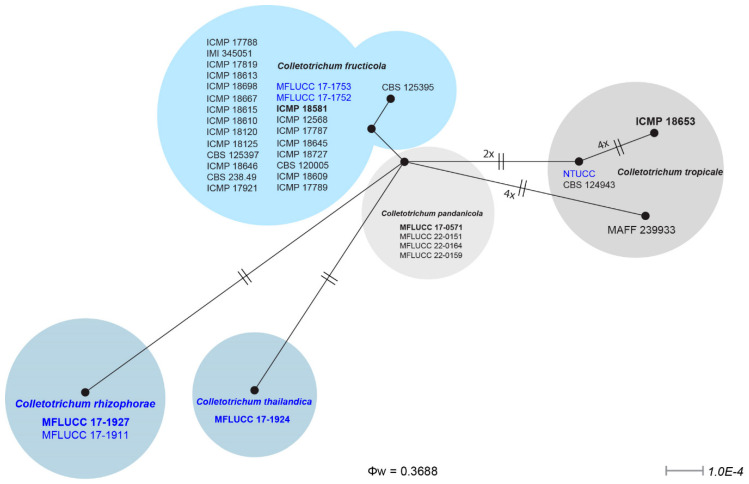
The results of the pairwise homoplasy index (PHI) test for closely related species of *Colletotrichum* stains in this study using both LogDet transformation and splits decomposition. PHI test results (Φw) > 0.05 indicate no significant recombination within the dataset.

To assess evolutionary independence, we employed the GCPSR concept on our strain dataset and its closely related taxa. The pairwise homoplasy index (PHI or Φw) is a crucial metric, and a value below 0.05 suggests the presence of substantial genetic recombination within a dataset. Figure 2 shows that our GCPSR analysis gave a PHI of 0.3688 for all closely related taxa in this study. This means that there was no significant genetic mixing between these strains and their sister taxa. Since we saw that the newly introduced species were very different from each other in terms of their phylogeny, we extended the GCPSR analysis to isolate only these new species. The results showed that the PHI value was greater than 0.05 (Φw = 1.0) for both newly taxon *C. rhizophorae* and *C. thailandica* isolates with the known species *C. pandanicola*. This clearly shows that these two new species have not been recombined in a significant way. This substantiates the distinct species status of all these isolates.

### 3.1. Colletotrichum fructicola Prihast., L. Cai and K.D. Hyde, Fungal Diversity 39: 96 (2009)

**Figure 3:**
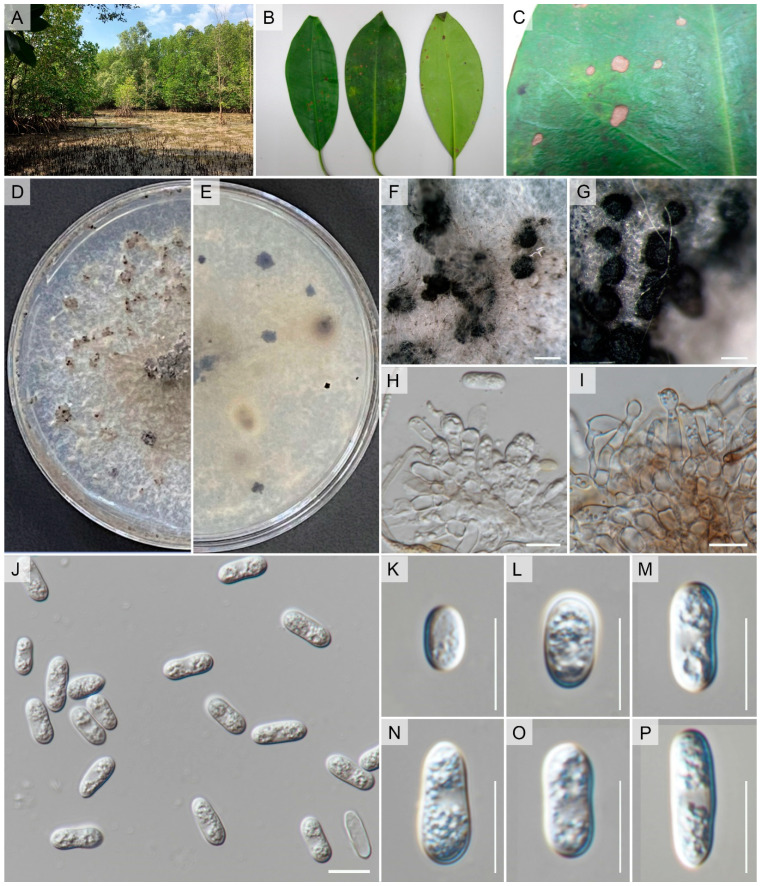
*Colletotrichum fructicola* (MFLUCC 17-1752). (**A**) Habitat. (**B**,**C**) *Rhizophora apiculata* leaf spot. (**D**) Culture on CMA (leaf-above, right-reverse). (**E–G**) Conidiomata on PDA. (**H**,**I**) Conidiogenous cells giving rise to conidia. (**J–P**) Conidia. Scale bars: (**F**) = 200 µm, (**G**) = 500 µm, (**H**–**P**) 10 µm.

**Figure 4:**
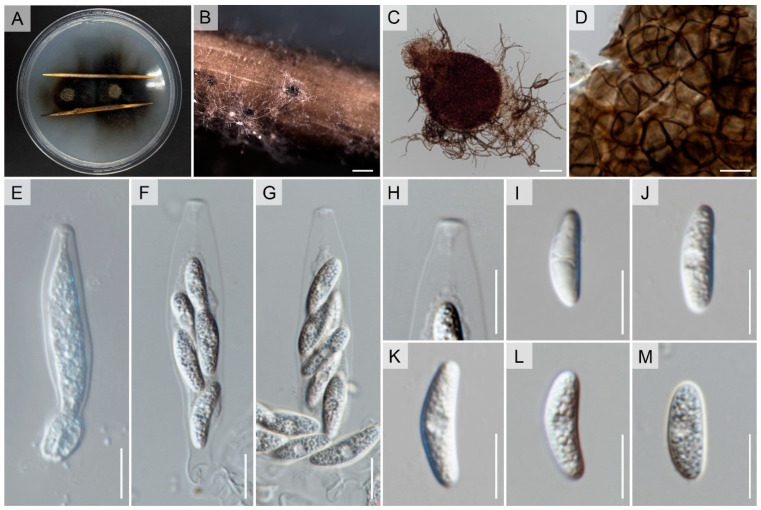
Sexual morph of *Colletotrichum fructicola* (MFLUCC 17-1752)**.** (**A**) Culture on WA with sterilized sticks. (**B**) Ascomata habitat on sterilized sticks. (**C**) Ascoma. (**D**) Ascoma peridium. (**E**–**G**) Immature and mature asci. (**H**) Apical ring in Melzer’s reagent. (**I**–**M**) Ascospores. Scale bars: (**B**) = 100 µm, (**C**) = 50 µm, (**D**–**M**) = 10 µm.

*Faces of Fungi number*: FoF 06767, Figure 3 and Figure 4

Isolated from the leaf spot that is associated with *Rhizophora apiculata* Blume. Asexual morph: *Conidiomata* pycnidial, globose, brown, superficial on PDA, releasing conidia in a yellow mass, slimy, globose, glistening mass. *Conidiophores* either directly formed from hyphae or from a cushion of spherical hyaline cells, septate, branched. *Conidiogenous cells* hyaline, cylindrical to ampulliform, straight to flask-shaped, 5–15 × 3–5 μm. *Setae* not observed. *Conidia* (9–)12.5–13(–14) × (4.7–)4–5(–5.5) μm (mean ± SD = 13 ± 0.5 × 5 ± 0.5 μm), hyaline, aseptate, smooth-walled, clavate to cylindrical, one end rounded and one end acute or both ends rounded, guttulate, granular. Sexual morph: *Ascomata* pycnidial, produced on WA + needle, sub-globose with ostiole, superficial, brown. Asci 30–68 × 8–14 μm, clavate to cymbiform, slightly curved, composed of pale to medium brown flattened angular cells, bitunicate, smooth-walled, 6–8-spored, with visible apical chamber, hyaline. *Ascospore* (14–)15–16(–19) × (4.4–)4–5(–5.3) μm (mean ± SD = 16 ± 0.5 × 5 ± 0.5 μm), hyaline, aseptate, smooth-walled, allantoid to lunate, both ends rounded, guttulate, granular.

Culture characteristics: Colonies on CMA reaching 7–8 cm diam after 7 d at room temperature (±25 °C), under light 12 h/dark 12 h, colonies rhizoid to filamentous, dense, flat or raised surface, with filiform margin, white from above and white to pale-yellow reverse, with producing grouped pycnidia. Colonies on WA with sterilized sticks, reaching 5 cm diam after 7 d at room temperature (±25 °C), under light 12 h/dark 12 h, colonies rhizoid to filamentous, dense, flat surface, with filiform margin, dark green from above and reverse, with producing pycnidia on sticks and immersed pycnidia under media.

*Hosts and Distribution*: *Actinidia chinensis*, China [56], Japan [57]; *Aesculus chinensis*, China [58]; *Amomum villosum*, China [59]; *Anacardium occidentale*, Brazil [60,61]; *Annona* spp., Brazil [62,63]; *Anthurium*, Sri Lanka [64]; *Arachis hypogaea*, China [65]; *Areca catechu*, China [66,67]; *Atractylodes ovata*, Korea [68]; *Aucuba japonica*, China [69], Korea [70]; *Averrhoa carambola*, China [71]; *Bletilla striata*, China [72]; *Brassica* spp., China [73]; *Camellia chrysantha*, China [74]; *Camellia grijsii*, China [75,76]; *Camellia oleifera*, China [77]; *Camellia sinensis*, China [67,78,79,80,81,82,83], Indonesia [42,80]; *Capsicum* spp., China [84,85], Thailand [86]; *Carica papaya*, Mexico [87]; *Carya* spp., China [88,89,90,91]; *Cattleya* spp., Brazil [92]; *Ceanothus thyrsiflorus*, Italy [93]; *Citrus* spp., China [19,94]; *Coffea arabica*, Thailand [32,42], China [95]; *Corchorus* sp., China [96,97]; *Cunninghamia lanceolata*, China [98,99]; *Curcuma phaeocaulis*, China [100]; *Cyclamen* sp., Italy [93]; *Cymbopogon citratus*, Thailand [101,102]; *Dalbergia hupeana*, China [103]; *Dendrobium* spp. China, Thailand [104,105]; *Dimocarpus longan*, Thailand [101]; *Dioscorea* spp., Nigeria [42]; *Diospyros kaki*, Brazil [106], China [107,108],; *Eichhornia crassipes*, China [109]; *Eriobotrya japonica*, China [110]; *Eucalyptus* spp., [111]; *Ficus edulis*, Germany [42]; *Fragaria* × *ananassa*, China [112,113,114,115,116]; *Glycine max*, China [117]; *Hedera* spp., China [100,118,119]; *Hevea brasiliensis*, China [120]; *Hydrangea paniculate*, Italy [93]; *Ilex chinensis*, China [100]; *Illicium verum*, China [121]; *Juglans regia*, China [122,123]; *Licania tomentosa*, Brazil [124]; *Ligustrum lucidum*, China [100]; *Limonium* sp., Israel [42]; *Liquidambar styraciflua*, Italy [93]; *Liriodendron* spp., China [125]; *Loropetalum chinense*, China [126]; *Luffa cylindrica*, China [127]; *Macadamia ternifolia*, China [128]; *Magnolia garrettii*, China [27]; *Magnolia* spp., China [129,130,131,132]; *Malus domestica*, USA [42,133], Brazil [42,134,135,136], Uruguay [135,137,138,139], China [140,141], Korea [142,143,144,145], Japan [146], Italy [147] and France [148]; *Mangifera indica*, Brazil, Mexico, Egypt, China, Korea, India [20,25,149,150,151,152,153,154,155]; *Manihot esculenta*, China [156], Brazil [157,158]; *Morus alba*, China [159]; *Musa* spp., China [160]; *Myrica rubra*, China [161]; *Nephelium lappaceum*, Puerto Rico [162]; *Nicotiana tabacum*, China [163]; *Nopalea cochenillifera*, Brazil [164]; *Osmanthus fragrans*, China [30]; *Paris polyphylla*, China [165,166]; *Pennisetum purpureum*, Thailand [102]; *Persea americana*, New Zealand [167], Australia [42], China [168], Israel [169], Colombia [170], Mexico [171,172]; *Peucedanum praeruptorum*, China [173]; *Phalaenopsis* sp., Brazil [92]; *Phoebe sheareri*, China [174]; *Pouteria caimito*, China [175]; *Prunus persica*, USA [176,177], China [178], Korea [179]; *Prunus salicina*, China [180,181,182]; *Pyrus* spp., China [42,183,184,185], and Korea [186]; *Radermachera sinica*, China [187]; *Rhizophora apiculate*, Thailand (in this study); *Rubus* spp., Colombia [188], China [189]; *Salvia greggii*, Italy [190]; *Selenicereus undatus*, Thailand [191]; *Tetragastris panamensis*, Panama [42]; *Tetrapanax papyrifer*, China [192]; *Theobroma cacao*, Panama [42]; *Vitis* spp., Korea [193], Brazil [194]; *Zamia furfuracea*, China [195]; *Zingiber officinale*, China [100]; *Ziziphus jujuba*, China [196,197,198]; *Ziziphus* sp., Thailand [191]; Human, China [199]; Nematodes, Worms *Chordodes formosanus*, China [200].

*Material examined*: Thailand, Chanthaburi Province, associated on leaf spot of *Rhizophora apiculata*, 25 April 2017, Kevin D. Hyde JT04-1, living cultures, MFLUCC 17-1752 (dried culture in MFLU 23-0476); JT04-2, living cultures, MFLUCC 17-1753 (dried culture in MFLU 23-0477).

*Notes:* Based on samples taken from *Coffea arabica* in Thailand, Prihastuti et al. [201] described *Colletotrichum fructicola* (Figure 3 and Figure 4). This taxon has various ecological roles, including epiphytic, endophytic, and pathogenic associations [202]. Yang et al. [203], which summarized subsequent research, showed *Colletotrichum fructicola* has a widespread distribution across a variety of host species. Through single and combined-gene phylogenetic analysis, our strain consistently grouped with *C. fructicola*, a species within the gloeosporioides species complex. This alignment was observed in both the preliminary analysis of the *Colletotrichum* species complex dataset and Figure 1. Furthermore, our strain exhibited morphological characteristics similar to *C. fructicola*, such as conidia size (in our study; 13.2 ± 0.5 × 5 ± 0.3 μm versus to 9.7–14 × 3–4.3 μm: Prihastuti et al. [201]), ascus size (in our study; 30–68 × 8–14 μm versus 30–55 × 6.5–8.5 μm: Prihastuti et al. [201]), featuring clavate to cymbiform asci, and ascospores (in our study; 16 ± 0.5 × 5 ± 0.5 μm versus to 9–14 × 3–4 μm: Prihastuti et al. [201]), which were hyaline and lunate. Consequently, we classify our strain as *C. fructicola*. This is the first record of an endophytic *C. fructicola* isolated from *Rhizophora apiculata* in Thailand.

### 3.2. Colletotrichum rhizophorae Norph. and K.D. Hyde sp. nov.

**Figure 5:**
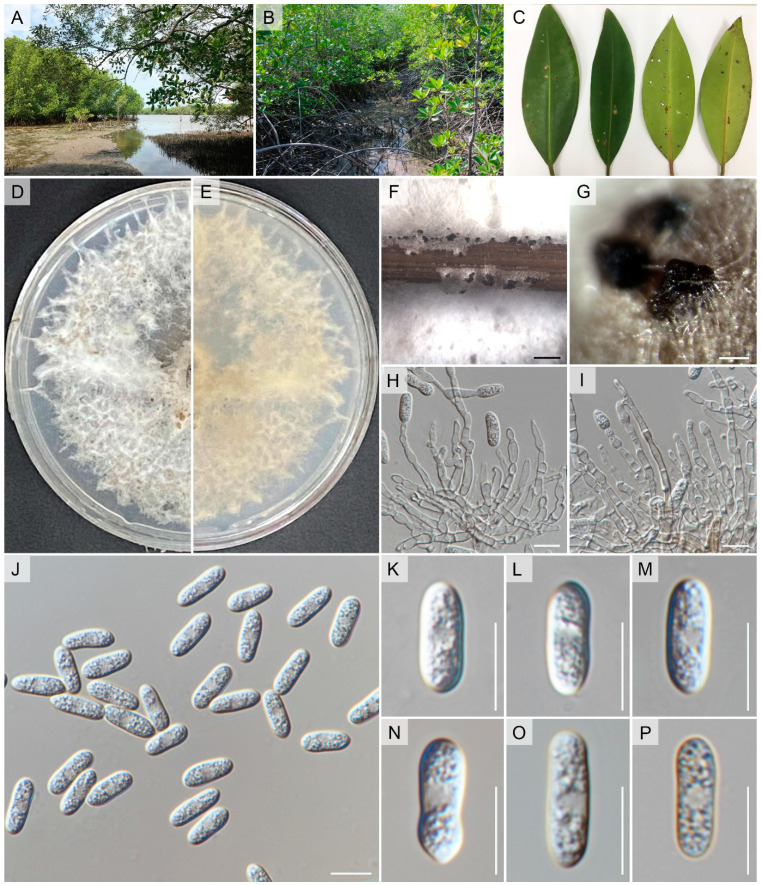
*Colletotrichum rhizophorae* (MFLUCC 17-1927). (**A**) Habitat. (**B**,**C**) *Rhizophora apiculata*. (**D**,**E**) Culture on PDA (leaf-above, right-reverse). (**F**) conidiomata on WA with sterilized sticks. (**G**) Conidiomata on PDA. (**H**,**I**) Conidiogenous cells giving rise to conidia. (**J**–**P**) Conidia. Scale bars: (**F**) = 200 µm, (**G**) = 500 µm, (**H**–**P**) 10 µm.

Index Fungorum number: IF901452; Faces of Fungi number: FoF 14890, Figure 5
Etymology: refers to the host from which the fungus was isolated, *Rhizophora apiculata* Blume.
Holotype: MFLU 23-0478

Isolated from an asymptomatic leaf spot of *Rhizophora apiculata* Blume. Sexual morph: undetermined. Asexual morph: *Conidiomata* pycnidial, globose, dark brown, superficial on PDA, releasing conidia in a yellow mass, slimy, globose. *Conidiophores* either directly formed from hyphae or from a cushion of spherical hyaline cells, septate, branched. *Conidiogenous cells* hyaline to pale brown, cylindrical to clavate, straight to flask-shaped, 6–19 × 2–9 μm. *Setae* not observed. *Conidia* (11.5–)12.5–13(–14.5) × (4–)4.5–5(–5.7) μm (mean ± SD = 13.1 ± 0.9 × 4.5 ± 0.3 μm), hyaline, aseptate, smooth-walled, ellipsoidal to cylindrical, one end rounded and one end acute or both ends rounded, guttulate, granular.

*Culture characteristics*: Colonies on PDA reaching 6–7 cm diam after 7 d at room temperature (±25 °C), under light 12 h/dark 12 h, colonies filamentous to circular, medium dense, aerial mycelium on surface flat, with irregular margin, white from above and reverse, with producing pycnidia and yellow spore mass.

Distribution: Thailand

Hosts: *Rhizophora apiculata*

*Material examined*: Thailand, Wan Yao, Khlung, Chanthaburi, asymptomatic leaf of *Rhizophora apiculata*, 25 April 2017, Kevin D. Hyde WYKE04AP (dried culture, MFLU 23-0478, holotype), living cultures, MFLUCC 17-1927; WYKE04AL, MFLUCC 17-1911 (dried culture MFLU 23-0479).

**Figure 6:**
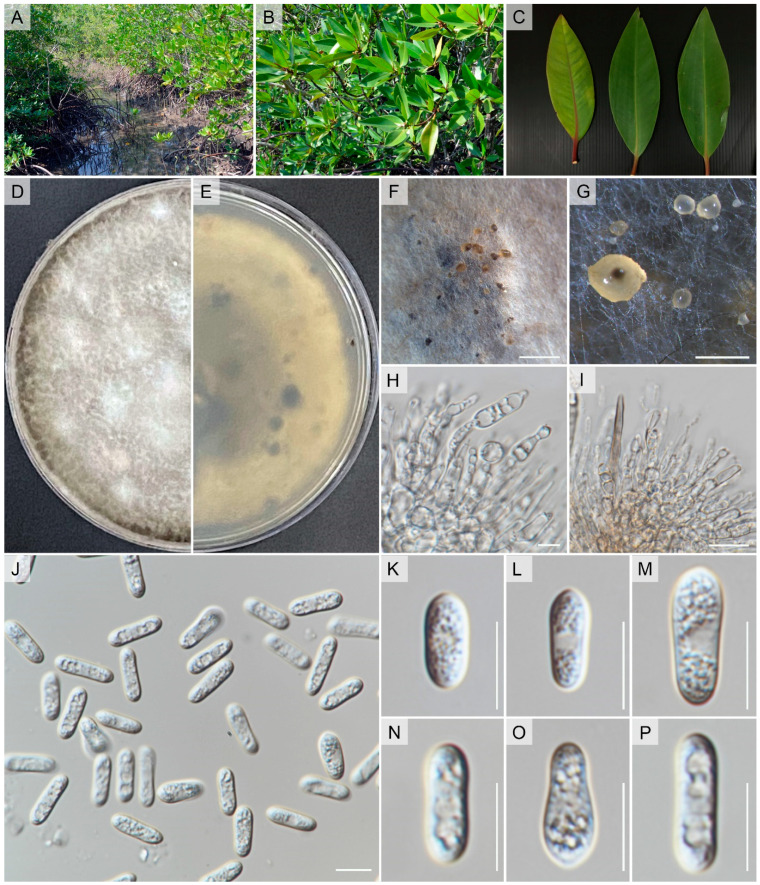
*Colletotrichum thailandica* (MFLUCC 17-1924)**.** (**A**) Habitat. (**B**,**C**) *Rhizophora apiculata*. (**D**,**E**) Culture on PDA (leaf-above, right-reverse). (**F**,**G**) Conidiomata on PDA. (**H**) Conidiogenous cells giving rise to conidia. (**I**) Setae. (**J**–**P**) Conidia. Scale bars: (**F**) = 250 µm, (**G**) = 500 µm, (**H**–**P**) 10 µm.

*Notes*: We introduce *Colletotrichum rhizophorae* as a novel species discovered within *Rhizophora apiculata*, a mangrove plant in Thailand (Figure 5). This classification is supported by morphological and phylogenetic evidence, as depicted in Figure 1. The phylogenetic analysis demonstrates that this new taxon closely associates with *C. thailandica* (Figure 1). However, notable distinctions in morphology are observed between *C. rhizophorae* and *C. thailandica*, particularly in conidia, conidiophores, and conidiogenous cells (refer to Figure 5 and Figure 6). In order to establish evolutionary independence, we applied the GCPSR concept to *C. rhizophorae* and its neighboring taxa. Our dataset yielded a PHI value exceeding 0.05 (Φw = 0.363), indicating the absence of significant genetic recombination between *C. rhizophorae* and its sister taxa, namely *C. pandanicola* and *C. thailandica* (Figure 2). Furthermore, a comparison of nucleotide sequences within ITS, *act*, *gapdh*, *β-tubulin*, *chs-1*, and *SCDgle* revealed discrepancies between *C. thailandica* and *C. rhizophorae* (ITS 5 bp, *act* 3 bp, *gapdh* 4 bp, *β*-*tubulin* 2 bp, *chs-1* 6 bp, and *SCDgle* 4 bp).

### 3.3. Colletotrichum thailandica Norph. and K.D. Hyde sp. nov.

Index Fungorum number: IF901453; Faces of Fungi number: FoF 14891, Figure 6
Etymology: refers to the country where the fungus was collected, Thailand.
Holotype: MFLU 23-0480

*Isolated* from an asymptomatic leaf spot of *Rhizophora apiculata* Blume. Sexual morph: undetermined. Asexual morph: *Conidiomata* pycnidial, globose, dark brown, superficial on PDA, releasing conidia in a yellow mass, slimy, globose, glistening mass. *Conidiophores* either directly formed from hyphae or from a cushion of spherical hyaline cells, septate, branched. *Conidiogenous cells* hyaline to pale brown, cylindrical to ampulliform, straight to flask-shaped, 6–16 × 2–5 μm. *Setae* about 40–85 µm long, brown to pale brown, and septate. *Conidia* (12.3–)13.5–15.5(–17.4) × (3.8–)4–4.5(–5.3) μm (mean ± SD = 14.7 ± 1.2 × 4 ± 0.3 μm), hyaline, aseptate, smooth-walled, clavate to cylindrical, one end rounded and one end acute or both ends rounded, guttulate, granular.

*Culture characteristics*: Colonies on PDA reaching 7–8 cm diam after 10 d at room temperature (±25 °C), under light 12 h/dark 12 h, colonies filamentous to circular, medium dense, aerial mycelium on surface flat or raised, with filiform margin (curled margin), fluffy, white from above and white to pale-yellow reverse, with producing pycnidia and yellow spore mass.

Distribution: Thailand.

Hosts: *Rhizophora apiculate*

*Material examined*: Thailand, Wan Yao, Khlung, Chanthaburi, asymptomatic leaf of *Rhizophora apiculata*, 25 April 2017, Kevin D. Hyde WYKE07AL, Living Cultures, MFLUCC 17-1924 (dried culture MFLU).

*Notes*: Thailand, Wan Yao, Khlung, Chanthaburi, asymptomatic leaf of *Rhizophora apiculata*, 25 April 2017, Kevin D. Hyde WYKE07AL (dried culture MFLU 23-0480, holotype), living cultures, MFLUCC 17-1924.

*Notes*: *Colletotrichum thailandica* is introduced here as a new species in the gloeosporioides species complex, a classification supported by both morphological (Figure 6) and phylogenetic data. The phylogenetic analysis underscores the distinctiveness of this new taxon, clearly separating it from other recognized *Colletotrichum* species (Figure 1).

In order to assess evolutionary autonomy, we applied the GCPSR concept to *C. thailandica* and its closely related taxa. Our data showed that the PHI value was higher than 0.05 (Φw = 0.363), which means that there was not much genetic mixing between *C. thailandica* and its closest relatives, *C. pandanicola* and *C. rhizophorae* (Figure 2). Since there was a lot of phylogenetic diversity between newly introduced species and species that had already been published, like *C. pandanicola*, we used GCPSR analysis on a larger dataset. The outcome revealed a PHI value surpassing 0.05 (Φw = 1.0), unequivocally indicating the absence of significant recombination for this new species. As a result, we formally introduce *C. thailandica* as a distinct species, isolated from *Rhizophora apiculata* in Thailand.

### 3.4. Colletotrichum tropicale E.I. Rojas, S.A. Rehner & Samuels, Mycologia 102(6): 1331 (2010)

**Figure 7:**
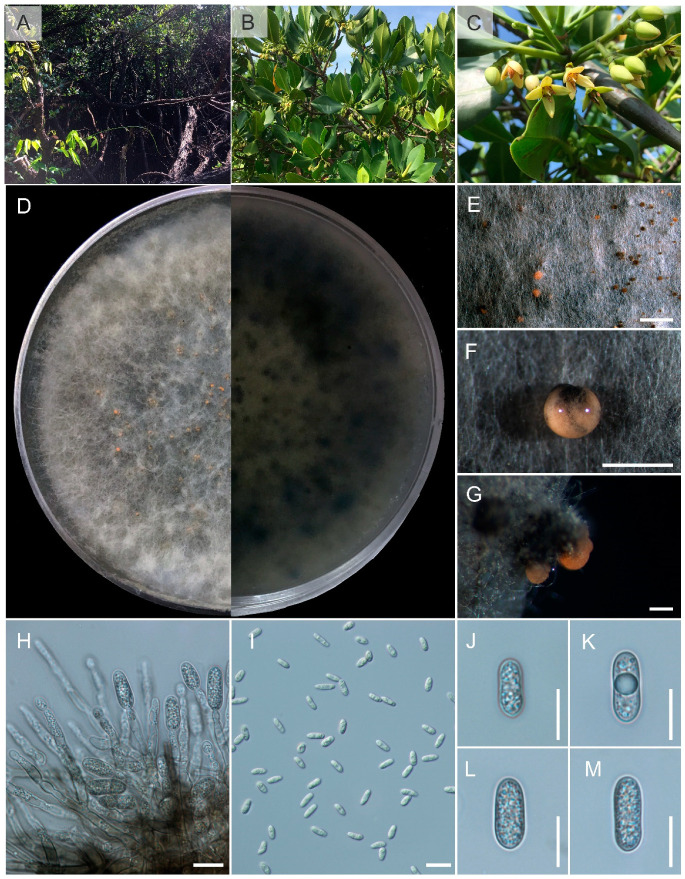
*Colletotrichum tropicale* (NCYU). (**A**) Habitat. (**B**,**C**) *Rhizophora mucronata*. (**D**) Culture on PDA (leaf-above, right-reverse). (**E**–**G**) Colony sporulating on PDA. (**H**) Conidiogenous cells giving rise to conidia. (**I**–**M**) Conidia. Scale bars: (**E**) = 2 mm, (**F**) = 100 µm, (**G**) = 50 µm, (**H**,**M**) = 10 µm, (**J**–**L**) = 20 µm.

*Faces of Fungi number*: FoF 14892, Figure 7

*Isolated* from the asymptomatic leaf of *Rhizophora mucronata* Lam. Sexual morph: undetermined. Asexual morph: *Conidiomata* pycnidial, globose, brown, superficial on PDA, releasing conidia in a yellow mass, slimy, globose, glistening mass. *Conidiophores* either directly formed from hyphae or from a cushion of spherical hyaline cells, septate, branched. *Conidiogenous cells* hyaline to pale brown, cylindrical to ampulliform, straight to flask-shaped, 10–20 × 3–5 μm. *Conidia* (12–)12.5–13(–14) × (4–)4.5–5(–5.7) μm (mean ± SD = 13.2 ± 0.5 × 5 ± 0.3 μm), hyaline, aseptate, smooth-walled, clavate to cylindrical, one end rounded and one end acute or both ends rounded, guttulate, granular.

*Culture characteristics*: Colonies on PDA reaching 7–8 cm diam after 14 d at room temperature (±25 °C), under light 12 h/dark 12 h, colonies filamentous to circular, medium dense, aerial mycelium on surface flat or raised, with filiform margin (curled margin), fluffy, gravy from above and dark gravy reverse, with producing pycnidia and yellow spore mass (Figure 3D).

*Hosts and distribution*: *Anacardium*, Brazil [60,61]; *Annona* spp., Brazil, Colombia, Panama and Cuba [63,204,205,206]; *Capsicum* spp., Indonesia [86] and Brazil [207]; *Carica papaya*, Costa Rica [208]; *Cariniana legalis*, Brazil [209]; *Cattleya* spp., Brazil [92]; *Cenchrus purpureus* [102]; *Coffea* spp., China [95]; *Copernicia prunifera*, Brazil [210]; *Cordia alliodora*, Panama [206]; endophyte *Trichilia tuberculata*, Panama [206]; *Ficus* spp., China [211,212]; *Licania tomentosa*, Brazil [124]; *Litchi chinensis*, Japan [42]; *Malpighia emarginata*, Japan [213]; *Mangifera indica* Brazil, Mexico, China [20,151,152,153,155,214]; *Manihot* spp., Brazil [215]; *Musa* spp., Brazil [216]; *Myrciaria dubia*, Brazil [217]; *Nelumbo nucifera*, China [218]; *Origanum vulgare*, Mexico [219]; *Passiflora edulis*, Brazil [220]; *Persea americana*, Mexico [172]; *Plinia cauliflora*, Japan [221]; *Punica granatum*, Brazil [222]; *Rhizophora mucronata*, Taiwan (in this study); *Sauropus androgynus*, China [223]; *Selenicereus monacanthus*, Philippines [224], Mexico [225]; *Theobroma cacao*, Panama [206]; *Viola surinamensis*, Panama [206]; Human [199].

*Material examined*: China, Taiwan, Tainan, Shicao, tissue isolation from asymptomatic leaves of *Rhizophora mucronata*, 17 July 2018, Chada Norphanphoun SCE3L-3B; living cultures, NTUCC.

*Notes*: *Colletotrichum tropicale* was documented by Rojas et al. [206] based on isolates obtained from *Theobroma cacao* leaves in Panama (Figure 7). This taxon has various ecological roles, including epiphytic, endophytic, and pathogenic with wide hosts and distribution [202]. The species was recorded as an endophyte in tropical regions associated with *Annona muricata* (Annonaceae), *Cenchrus purpureus* (Poaceae), *Cordia aliodora* (Boraginaceae), *Cymbopogon citratus* (Poaceae), *Litchi chinensis* (Sapindaceae), *Nelumbo nucifera* (Nelumbonaceae), *Theobroma cacao* (Malvaceae), *Trichilia tuberculata* (Meliaceae), *Viola surinamensis* (Myristicaceae) [42,102,206,218]. In our current study, our phylogenetic analysis clearly places our strain within the *C. tropicale* clade with robust support, as illustrated in Figure 1. This grouping is further substantiated by the striking similarity in conidia morphology and size, as observed in our study (13.2 ± 0.5 × 5 ± 0.3 μm) when compared to the reported values by Rojas et al. [206] in 2010 (14.1–14.8 × 5.1–5.20 μm). Consequently, we formally designate our isolate as *C. tropicale*, representing the first documented instance of an endophytic fungus isolated from *Rhizophora mucronata* in Taiwan.

## 4. Discussion

*Colletotrichum* is a pathogenic genus that affects various plant species, including mangroves [28,29,30]. It causes anthracnose, a common disease characterized by dark lesions on leaves, stems, and fruits [16]. Several studies have investigated the prevalence and impact of *Colletotrichum* on mangroves, providing valuable data for understanding its ecology and management strategies [28,35,36]. In this study, we focused on the examination of six strains isolated from mangrove ecosystems. Five of these strains were isolated from *Rhizophora apiculata* in Thailand’s mangroves, while one strain originated from *Rhizophora mucronata* in Taiwan. Among the isolates, *Colletotrichum fructicola* (MFLUCC 17-1752) was obtained from leaf spot symptoms, while the remaining strains were isolated from asymptomatic leaves. It is important to note that *C. fructicola* has been found to play different roles in the environment, including as an epiphyte, an endophyte, and a pathogen in a wide range of host species [203]. This suggests that the presence of *Colletotrichum* species in mangrove ecosystems may be more diverse than initially anticipated. These taxa can exhibit various ecological interactions, including their colonization of asymptomatic leaves. As a result, there is potential for the discovery of additional fungal species within mangrove forest zones. These newly discovered species could encompass both those commonly found in other plant species and entirely novel fungal types.

This comprehensive study employed phylogenetic analysis, morphological characterization, and the Genetic Clade–Phenetic Species Recognition (GCPSR) concept to elucidate the taxonomy and evolutionary relationships of these *Colletotrichum* species within the gloeosporioides species complex according to the guidelines of Chethana et al. [226] and Maharachchikumbura et al. [227]. Previously, Weir et al. [42] documented the efficacy of individual genes in discerning species within the gloeosporioides species complex. The study identified the designated barcoding gene for fungi in the gloeosporioides complex, encompassing eight genes: the internal transcribed spacer region (ITS), actin (*act*), glyceraldehyde-3-phosphate dehydrogenase region (*gapdh*), beta-tubulin (*β-tubulin*), chitin synthase (*chs-1*), calmodulin (*cal*), glutamine synthetase (*GS*), and manganese-superoxide dismutase (*SOD2*). However, it was observed that these genes do not consistently provide a conclusive resolution of relationships for all species within this particular species complex. In the context of *C. siamense*, the performance of individual genes that can distinguish species within the *C. gloeosporioides* species complex is notably achieved by examining *cal* and *β-tubulin* sequences. Conversely, for *C. tropicale*, the distinguishing genes encompass *β-tubulin*, *act*, *GS*, and *SOD2*. In the case of *C. fructicola*, the pertinent genes for effective differentiation are *cal*, *chs-1*, *GS*, and *SOD2*. To overcome limitations associated with gene function in species delimitation and to achieve precise identification of *Colletotrichum* isolates in the present study, a comprehensive approach employing six gene sequences (ITS, *act*, *gapdh*, *β-tubulin*, *chs-1*, and *cal*), encompassing 126 strains, and 2 singleton strains as outgroups were used to facilitate the identification of two novel species and to document a new host record from Thailand. Moreover, there is a new record of *C. tropicale* from Taiwan, using *act*, *β-tubulin*, and *chs-1*. The study encompasses multiple reference isolates of *C. fructicola*, *C. siamense*, and *C. tropicale*. The results of a multigene phylogenetic analysis demonstrated that the combined use of ITS, *act*, *gapdh*, *β-tubulin*, *chs-1*, and *cal* offered superior resolution in determining *Colletotrichum* species, surpassing the efficacy of single-gene analysis. This finding aligns with prior studies conducted by Prihastuti et al. [201] and Weir et al. [42]. The results provided valuable insights into the diversity and classification of *Colletotrichum* species. The phylogenetic analysis, utilizing both maximum likelihood (ML) and Bayesian inference (BI) methods, revealed a well-supported clustering of the new strains within the gloeosporioides species complex clade, alongside sequences previously identified as members of this complex. The robust statistical support, with 100% bootstrap support (BSML) and a posterior probability of 1.00 (PPBI), underlined the validity of the species complex classification (Figure 1). Within this complex, two novel species are formally recognized: *C. rhizophorae* and *C. thailandica*. These designations were supported by a high bootstrap support of 99% (BSML) and a posterior probability of 1.00 (PPBI), reaffirming their distinct species status. Additionally, known species, including *C. fructicola* and *C. tropicale*, were identified and validated based on their placement within the phylogenetic tree. The application of the GCPSR concept further corroborated the evolutionary independence of these species. The pairwise homoplasy index (PHI or Φw) values exceeding 0.05 indicated a lack of significant genetic recombination within the dataset, highlighting the distinctiveness of the newly proposed species. This was particularly evident in the case of *C. rhizophorae* and *C. thailandica*, as their PHI values exceeded 0.05 even when analyzed with closely related taxa. The study delved into the taxonomy of two *Colletotrichum* species, *C. fructicola* and *C. tropicale*, offering significant insights into their classification, morphology, and distribution.

*Colletotrichum fructicola*, originally described in 2009 from *Coffea arabica* in Thailand [201], was the subject of taxonomic reevaluation. The study consistently found that the strain under investigation clustered closely with known *C. fructicola* strains within the gloeosporioides species complex. This clustering was observed both in the preliminary analysis and the final phylogenetic tree, reaffirming its placement within this species complex. Furthermore, morphological similarities, including conidia size, asci size, and ascospore features, provided additional support for the classification of the strain as *C. fructicola*. Importantly, this study marked a significant milestone in scientific discovery by documenting the first-ever instance of an endophytic fungus isolated from *R. apiculata* in Thailand.

*Colletotrichum tropicale*, initially documented from *T. cacao* leaves in Panama [206], was also investigated in this study. The research employed phylogenetic analysis and examination of conidia morphology to validate the classification of the study’s isolate as *C. tropicale*. This confirmation represented a notable scientific contribution, as it marked the first documented instance of an endophytic fungus isolated from *R. mucronata* in Taiwan.

These records expand our knowledge of the geographic distribution of these fungal species. In conclusion, this research enhances our understanding of fungal diversity within mangrove ecosystems and provides valuable taxonomic and ecological insights. The combined use of molecular, morphological, and ecological data, as well as genetic recombination analysis, strengthens the credibility of the newly introduced *Colletotrichum* species. Overall, this research significantly contributes to our understanding of the taxonomy and evolutionary relationships within the *Colletotrichum* species complex. The combination of molecular, morphological, and ecological data has led to the recognition of novel species and the validation of known ones, enhancing our knowledge of these important plant-associated fungi.

## Figures and Tables

**Table 1:** Polymerase chain reaction (PCR) thermal cycling programs for each locus.

Gene	Primers	PCR Thermal Cycle Protocols *
ITS	ITS1/ITS4	ID 95 °C for 5 min, 40 cycles of D at 95 °C for 45 s, A at 53 °C for 45 s, E at 72 °C for 2 min, FE at 72 °C for 10 min
*actin*	ACT512F/ACT738R	ID 95 °C for 3 min, 35 cycles of D at 95 °C for 30 s, A at 56 °C for 30 s, E at 72 °C for 45 s, FE at 72 °C for 1 min
*gapdh*	GDF1/GPDHR2	ID 95 °C for 5 min, 35 cycles of D at 95 °C for 30 s, A at 50 °C for 45 s, E at 72 °C for 90 s, FE at 72 °C for 7 min
*β-tubulin*	T1/T2	ID 95 °C for 3 min, 35 cycles of D at 95 °C for 30 s, A at 543 °C for 30 s, E at 72 °C for 45 s, FE at 72 °C for 1 min
*chs-1*	CHS-79F/CHS-354R	ID 95 °C for 3 min, 35 cycles of D at 95 °C for 30 s, A at 59 °C for 30 s, E at 72 °C for 45 s, FE at 72 °C for 1 min
*cal*	CL1C/CL2C	ID 94 °C for 3 min, 40 cycles of D at 95 °C for 30 s, A at 57 °C for 80 s, E at 72 °C for 80 s, FE at 72 °C for 10 min

* ID: initial denaturation; D = denaturation; A = annealing; E = elongation; FE = final extension.

**Table 2:** GenBank accession numbers of the sequences used in phylogenetic analyses Figure 1.

Species	Strain	Host	Country	Accession Numbers					
				ITS	*act*	*gapdh*	*β-tubulin*	*chs-1*	*cal*
*C. aenigma*	ICMP 18608 ^T^	*Persea americana*	Israel	JX010244	JX009443	JX010044	JX010389	JX009774	JX009683
*C. aeschynomenes*	ICMP 17673 ^T^	*Aeschynomene virginica*	USA	JX010176	JX009483	JX009930	JX010392	JX009799	JX009721
*C. alatae*	ICMP 17919 ^T^	*Dioscorea alata*	India	JX010190	JX009471	JX009990	JX010383	JX009837	JX009738
*C. alienum*	ICMP 12071 ^T^	*Malus domestica*	New Zealand	JX010251	JX009572	JX010028	JX010411	JX009882	JX009654
*C. analogum*	CGMCC 3.16079 ^T^	*Ageratina adenophora*	China	OK030860	OK513599	OK513663	OK513629	OK513559	-
*C. aotearoa*	ICMP 18537 ^T^	*Coprosma* sp.	New Zealand	JX010205	JX009564	JX010005	JX010420	JX009853	JX009611
*C. arecacearum*	LC13850, MH0003 ^T^	*Arecaceae*	China	MZ595867	MZ664165	MZ664049	MZ673986	MZ799262	MZ799238
*C. arecacearum*	LC13851, MH0003-1	*Arecaceae*	China	MZ595868	MZ664166	MZ664050	MZ673987	MZ799263	MZ799239
*C. arecicola*	CGMCC 3.19667 ^T^	*Areca catechu*	China	MK914635	MK935374	MK935455	MK935498	MK935541	-
*C. artocarpicola*	MFLUCC 18-1167 ^T^	*Artocarpus heterophyllus*	Thailand	MN415991	MN435570	MN435568	MN435567	MN435569	-
*C. asianum*	ICMP 18580 ^T^	*Coffea arabica*	Thailand	JX010196	JX009584	JX010053	JX010406	JX009867	FJ917506
*C. asianum*	CMM4057	*Mangifera indica*	Brazil	KC329792	KC533747	KC517168	KC517278	-	-
*C. asianum*	C1646	*Mangifera indica*	Taiwan (China)	MK326570	MK462967	MK376935	-	MK347247	-
*C. asianum*	TL107	*Mangifera indica*	China	MF039845	MF039758	MF040776	MF039816	MF039787	-
*C. asianum*	ICMP 18696	*M. indica*	Australia	JX010192	JX009576	JX009915	JX010384	JX009753	JX009723
*C. asianum*	CMM4056	*Mangifera indica*	Brazil	KC329789	KC533720	KC517165	KC517277	-	-
*C. australianum*	UMC002 ^T^	*Citrus sinensis*	Australia	MG572138	MN442109	MG572127	MG572149	MW091987	-
*C. camelliae*	CGMCC 3.14925 ^T^	*Camellia sinensis*	China	KJ955081	KJ954363	KJ954782	KJ955230	MZ799255	KJ954634
*C. cangyuanense*	CGMCC 3.18969 ^T^	*Ageratina adenophora*	China	OK030864	OK513603	OK513667	OK513633	OK513563	-
*C. changpingense*	SA0016 ^T^	*Fragaria × ananass*	China	KP683152	KP683093	KP852469	KP852490	KP852449	-
*C. chiangmaiense*	MFLUCC 18-0945 ^T^	*Magnolia garrettii*	Thailand	MW346499	MW655578	MW548592	-	MW623653	-
*C. chrysophilum*	CMM4268 ^T^	*Musa* sp.	Brazil	KX094252	KX093982	KX094183	KX094285	KX094083	KX094063
*C. cigarro*	ICMP 18539 ^T^	*Olea europaea*	Australia	JX010230	JX009523	JX009966	JX010434	JX009800	JX009635
*C. clidemiae*	ICMP 18658 ^T^	*Clidemia hirta*	Hawaii	JX010265	JX009537	JX009989	JX010438	JX009877	JX009645
*C. cobbittiense*	BRIP 66219 ^T^	*Cordyline stricta × C. australis*	Australia	MH087016	MH094134	MH094133	MH094137	MH094135	-
*C. conoides*	CGMCC 3.17615 ^T^	*Capsicum* sp.	China	KP890168	KP890144	KP890162	KP890174	KP890156	KP890150
*C. cordylinicola*	ICMP 18579 ^T^	*Cordyline fruticosa*	Thailand	JX010226	JX009586	JX009975	JX010440	JX009864	HM470238
*C. cycadis*	BRIP 71326a ^T^	*Cycas revoluta*	China	MT439915		MT439919	MT439921	MT439917	-
*C. dimorphum*	CGMCC 3.16083 ^T^	*Ageratina adenophora*	China	OK030867	OK513606	OK513670	OK513636	OK513566	-
*C. dracaenigenum*	MFLUCC 19-0430 ^T^	*Dracaena* sp.	Thailand	MN921250	MT313686	MT215577	-	MT215575	-
*C. endophyticum*	MFLUCC 13-0418 ^T^	*Pennisetum purpureum*	Thailand	KC633854	KF306258	KC832854	MZ673954	MZ799261	-
*C. fructicola*	ICMP 18581 ^T^	*Coffea arabica*	Thailand	JX010165	JX009501	JX010033	JX010405	JX009866	FJ917508
*C. fructicola*	ICMP 12568	*Persea americana*	Australia	JX010166	JX009529	JX009946	-	JX009762	JX009680
*C. fructicola*	ICMP 17787	*Malus domestica*	Brazil	JX010164	JX009439	JX009958	-	JX009807	JX009667
*C. fructicola*	ICMP 17788	*Malus domestica*	Brazil	JX010177	JX009458	JX009949	-	JX009808	JX009672
*C. fructicola*	IMI 345051, ICMP 17819	*Fragaria × ananassa*	Canada	JX010180	JX009469	JX009997	-	JX009820	JX009668
*C. fructicola*	ICMP 18613	*Limonium sinuatum*	Israel	JX010167	JX009491	JX009998	JX010388	JX009772	JX009675
*C. fructicola*	ICMP 18698	*Limonium* sp.	Israel	JX010168	JX009585	JX010052	-	JX009773	JX009677
*C. fructicola*	ICMP 18667	*Limonium* sp.	Israel	JX010169	JX009464	JX009951	-	JX009775	JX009679
*C. fructicola*	ICMP 18615	*Limonium* sp.	Israel	JX010170	JX009511	JX010016	-	JX009776	JX009678
*C. fructicola*	ICMP 18610	*Pyrus pyrifolia*	Japan	JX010174	JX009526	JX010034	-	JX009788	JX009681
*C. fructicola*	ICMP 18120	*Dioscorea alata*	Nigeria	JX010182	JX009436	JX010041	JX010401	JX009844	JX009670
*C. fructicola*	CBS 125395, ICMP 18645	*Theobroma cacao*	Panama	JX010172	JX009543	JX009992	JX010408	JX009873	JX009666
*C. fructicola*	ICMP 18727	*Fragaria × ananassa*	USA	JX010179	JX009565	JX010035	JX010394	JX009812	JX009682
*C. fructicola*	CBS 120005, ICMP 18609	*Fragaria × ananassa*	USA	JX010175	JX009534	JX009926	-	JX009792	JX009673
*C. fructicola*	ICMP 17789	*Malus domestica*	USA	JX010178	JX009451	JX009914	-	JX009809	JX009665
*C. fructicola*	ICMP 18125	*Dioscorea alata*	Nigeria	JX010183	JX009468	JX010009	-	JX009847	JX009669
*C. fructicola*	CBS 125397 ^T^, ICMP 18646	*Tetragastris panamensis*	Panama	JX010173	JX009581	JX010032	JX010409	JX009874	JX009674
*C. fructicola*	CBS 238.49 ^T^, ICMP 17921	*Ficus edulis*	Germany	JX010181	JX009495	JX009923	JX010400	JX009839	JX009671
***C. fructicola***	**MFLUCC 17-1752**	***Rhizophora apiculata***	**Thailand**	**OR828931**	**OR840845**	**OR840868**	**OR840862**	**OR840856**	**OR840851**
***C. fructicola***	**MFLUCC 17-1753**	***Rhizophora apiculata***	**Thailand**	**OR828932**	**OR840846**	**OR840869**	**OR840863**	**OR840857**	**OR840852**
*C. fructivorum*	CBS 133125 ^T^	*Vaccinium macrocarpon*	Burlington	JX145145	MZ664126	MZ664047	JX145196	MZ799259	-
*C. gloeosporioides*	ICMP 17821 ^T^	*Citrus sinensis*	Italy	JX010152	JX009531	JX010056	JX010445	JX009818	JX009731
*C. gracile*	CGMCC 3.16075 ^T^	*Ageratina adenophora*	China	OK030868	OK513607	OK513671	OK513637	OK513567	
*C. grevilleae*	CBS 132879 ^T^	*Grevillea* sp.	Italy	KC297078	KC296941	KC297010	KC297102	KC296987	KC296963
*C. grossum*	CGMCC 3.17614 ^T^	*Chili pepper*	China	KP890165	KP890141	KP890159	KP890171	KP890153	KP890147
*C. hebeiense*	MFLUCC 13-0726 ^T^	*Vitis vinifera*	China	KF156863	KF377532	KF377495	KF288975	KF289008	-
*C. hederiicola*	MFLU 15-0689 ^T^	*Hedera helix*	Italy	MN631384	MN635795	-	-	MN635794	-
*C. helleniense*	CBS 142418 ^T^	*Poncirus trifoliata*	Greece, Arta	KY856446	KY856019	KY856270	KY856528	KY856186	KY856099
*C. henanense*	CGMCC 3.17354 ^T^	*Camellia sinensis*	China	KJ955109	KM023257	KJ954810	KJ955257	MZ799256	KJ954662
*C. horii*	ICMP 10492 ^T^	*Diospyros kaki*	Japan	GQ329690	JX009438	GQ329681	JX010450	JX009752	JX009604
*C. hystricis*	CBS 142411 ^T^	*Citrus hystrix*	Italy, Catania	KY856450	KY856023	KY856274	KY856532	KY856190	KY856103
*C. jiangxiense*	CGMCC 3.17361 ^T^	*Camellia sinensis*	China	KJ955149	KJ954427	KJ954850	OK236389	MZ799257	KJ954701
*C. kahawae*	ICMP 17816 ^T^	*Coffea arabica*	Kenya	JX010231	JX009452	JX010012	JX010444	JX009813	JX009642
*C. makassarense*	CBS 143664 ^T^	*Capsicum annuum*	Indonesia	MH728812	MH781480	MH728820	MH846563	MH805850	-
*C. musae*	CBS 116870 ^T^	*Musa* sp.	USA	HQ596292	HQ596284	HQ596299	HQ596280	JX009896	JX009742
*C. nanhuaense*	CGMCC 3.18962 ^T^	*Ageratina adenophora*	China	OK030870	OK513609	OK513673	OK513639	OK513569	-
*C. nullisetosum*	CGMCC 3.16080 ^T^	*Mangifera indica*	China	OK030872	OK513611	OK513675	OK513641	OK513571	-
*C. nupharicola*	ICMP 18187 ^T^	*Nuphar lutea* subsp. *polysepala*	USA	JX010187	JX009437	JX009972	JX010398	JX009835	JX009663
*C. oblongisporum*	CGMCC 3.16074 ^T^	*Ageratina adenophora*	China	OK030874	-	OK513677	OK513643	OK513573	-
*C. pandanicola*	MFLUCC 17-0571 ^T^	Pandanaceae	Thailand	MG646967	MG646938	MG646934	MG646926	MG646931	-
*C. pandanicola*	MFLUCC 22-0164	Pandanaceae	Thailand	OP802369	OP801689	OP801724	OP801744	OP801706	-
*C. pandanicola*	MFLUCC 22-0151	Pandanaceae	Thailand	OP802371	OP801691	OP801726	OP801746	OP801708	-
*C. pandanicola*	MFLUCC 22-0159	Pandanaceae	Thailand	OP802373	OP801692	OP801727	OP801747	OP801709	-
*C. perseae*	CBS 141365 ^T^	*Avocado*	Israel	KX620308	KX620145	KX620242	KX620341	MZ799260	-
*C. proteae*	CBS 132882 ^T^	*Protea* sp.	South Africa	KC297079	KC296940	KC297009	KC297101	KC296986	KC296960
*C. pseudotheobromicola*	MFLUCC 18-1602 ^T^	*Prunus avium*	China	MH817395	MH853681	MH853675	MH853684	MH853678	-
*C. psidii*	ICMP 19120 ^T^	*Psidium* sp.	Italy	JX010219	JX009515	JX009967	JX010443	JX009901	JX009743
*C. queenslandicum*	ICMP 1778 ^T^	*Carica papaya*	Australia	JX010276	JX009447	JX009934	JX010414	JX009899	JX009691
*C. rhexiae*	CBS 133134 ^T^	*Rhexia virginica*	Sussex	JX145128	MZ664127	MZ664046	JX145179	MZ799258	-
***C. rhizophorae***	**MFLUCC 17-1927 ^T^**	***Rhizophora apiculata***	**Thailand**	**OR828933**	**OR840847**	**OR840870**	**OR840864**	**OR840858**	**OR840853**
***C. rhizophorae***	**MFLUCC 17-1911**	***Rhizophora apiculata***	**Thailand**	**OR828934**	**OR840848**	**OR840871**	**OR840865**	**OR840859**	**OR840854**
*C. salsolae*	ICMP 19051 ^T^	*Salsola tragus*	Hungary	JX010242	JX009562	JX009916	JX010403	JX009863	JX009696
*C. siamense*	ICMP 18578 ^T^	*Coffea arabica*	Thailand	JX010171	JX009518	JX009924	JX010404	JX009865	FJ917505
*C. siamense*	HSI-3	*Hymenocallis littoralis*	China	OM654563	OM831342	OM831360	OM831384	OM831354	-
*C. siamense*	ICMP 12567	*Persea americana*	Australia	JX010250	JX009541	JX009940	JX010387	JX009761	JX009697
*C. siamense*	DAR 76934, ICMP 18574	*Pistacia vera*	Australia	JX010270	JX009535	JX010002	JX010391	JX009798	JX009707
*C. siamense*	ICMP 12565	*Persea americana*	Australia	JX010249	JX009571	JX009937	-	JX009760	JX009698
*C. siamense*	CBS 125379, ICMP 18643	*Hymenocallis americana*	China	JX010258	GQ856776	JX010060	-	GQ856729	GQ849451
*C. siamense*	ICMP 18121	*Dioscorea rotundata*	Nigeria	JX010245	JX009460	JX009942	JX010402	JX009845	JX009715
*C. siamense*	ICMP 18117	*Dioscorea rotundata*	Nigeria	JX010266	JX009574	JX009954	-	JX009842	JX009700
*C. siamense*	ICMP 18739	*Carica papaya*	South Africa	JX010161	JX009484	JX009921	-	JX009794	JX009716
*C. siamense*	ICMP 18570	*Persea americana*	South Africa	JX010248	JX009510	JX009969	-	JX009793	JX009699
*C. siamense*	ICMP 18569	*Persea americana*	South Africa	JX010262	JX009459	JX009963	-	JX009795	JX009711
*C. siamense*	HKUCC 10884, ICMP 18575	*Capsicum annuum*	Thailand	JX010256	JX009455	JX010059	-	JX009785	JX009717
*C. siamense*	HKUCC 10881, ICMP 18618	*Capsicum annuum*	Thailand	JX010257	JX009512	JX009945	-	JX009786	JX009718
*C. siamense*	ICMP 18572	*Vitis vinifera*	USA	JX010160	JX009487	JX010061	-	JX009783	JX009705
*C. siamense*	ICMP 18571	*Fragaria × ananassa*	USA	JX010159	JX009482	JX009922	-	JX009782	JX009710
*C. siamense*	ICMP 17795	*Malus domestica*	USA	JX010162	JX009506	JX010051	JX010393	JX009805	JX009703
*C. siamense*	CBS 125378 (^T^), ICMP 18642	*Hymenocallis americana*	China	JX010278	GQ856775	JX010019	JX010410	GQ856730	JX009709
*C. siamense*	CBS 130420 (^T^), ICMP 19118	*Jasminum sambac*	Vietnam	HM131511	HM131507	HM131497	JX010415	JX009895	JX009713
*C. siamense*	ICMP 17785	*Malus domestica*	USA	JX010272	JX009446	JX010058	-	JX009804	JX009706
*C. siamense*	ICMP 18573	*Vitis vinifera*	USA	JX010271	JX009435	JX009996	-	JX009784	JX009712
*C. siamense*	ICMP 18118	*Commelina* sp.	Nigeria	JX010163	JX009505	JX009941	-	JX009843	JX009701
*C. siamense*	MFLUCC 22-0109	Pandanaceae	Thailand	OP740246	OP744511	OP744513	OP744514	OP744512	-
*C. siamense*	MFLUCC 22-0135	Pandanaceae	Thailand	OP802374	OP801693	OP801728	OP801748	OP801710	-
*C. siamense*	MFLUCC 22-0137	Pandanaceae	Thailand	OP802362	OP801686	OP801721	OP801740	OP801703	-
*C. siamense*	MFLUCC 22-0138	Pandanaceae	Thailand	OP802366	OP801688	OP801723	OP801742	OP801705	-
*C. siamense*	CGMCC 3.16078 ^T^	*Ageratina adenophora*	China	OK030876	OK513613	OK513679	OK513645	OK513575	-
*C. subhenanense*	CGMCC 3.16073 ^T^	*Ageratina adenophora*	China	OK030883	OK513618	OK513684	OK513647	OK513581	-
*C. syzygiicola*	MFLUCC 10-0624 ^T^	*Syzygium samarangense*	Thailand	KF242094	KF157801	KF242156	KF254880	-	KF254859
*C. tainanense*	CBS 143666 ^T^	*Capsicum annuum*	Taiwan (China)	MH728818	MH781475	MH728823	MH846558	MH805845	-
*C. temperatum*	CBS 133122 ^T^	*Vaccinium macrocarpon*	Bronx	JX145159	MZ664125	MZ664045	JX145211	MZ799254	-
*C. tengchongense*	YMF 1.04950, CGMCC 3.18950 ^T^	*Isoetes sinensis*	China	OL842169	OL981238	OL981264	-	OL981290	-
*C. theobromicola*	ICMP 18649 ^T^	*Theobroma cacao*	Panama	JX010294	JX009444	JX010006	JX010447	JX009869	JX009591
***C. thailandica***	**MFLUCC 17-1924 ^T^**	***Rhizophora apiculata***	**Thailand**	**OR828935**	**OR840849**	**OR840872**	**OR840866**	**OR840860**	**OR840855**
*C. ti*	ICMP 4832 ^T^	*Cordyline* sp.	New Zealand	JX010269	JX009520	JX009952	JX010442	JX009898	JX009649
*C. tropicale*	ICMP 18653 ^T^	*Theobroma cacao*	Panama	JX010264	JX009489	JX010007	JX010407	JX009870	JX009719
*C. tropicale*	MAFF 239933, ICMP 18672	*Litchi chinensis*	Japan	JX010275	JX009480	JX010020	JX010396	JX009826	JX009722
*C. tropicale*	CBS 124943, ICMP 18651	*Annona muricata*	Panama	JX010277	JX009570	JX010014	-	JX009868	JX009720
***C. tropicale***	**NTUCC**	***Rhizophora mucronata***	**Taiwan (China)**	**-**	**OR840850**	**-**	**OR840867**	**OR840861**	**-**
*C. viniferum*	CBS130643 ^T^	*Vitis vinifera* cv. *Shuijing*	China	JN412804	JN412795	JN412798	-	-	JQ309639
*C. vulgaris*	YMF 1.04940, CGMCC 3.18940 ^T^	*Hippuris vulgaris*	China	OL842170	OL981239	OL981265	-	OL981291	-
*C. wuxiense*	CGMCC 3.17894 ^T^	*Camellia sinensis*	China	KU251591	KU251672	KU252045	KU252200	KU251939	KU251833
*C. xanthorrhoeae*	ICMP 17903 ^T^	*Xanthorrhoea preissii*	Australia	JX010261	JX009478	JX009927	JX010448	JX009823	JX009653
*C. xishuangbannaense*	MFLUCC 19-0107 ^T^	*Magnolia liliifera*	China	MW346469	MW652294	MW537586	-	MW660832	-
*C. yuanjiangense*	CGMCC 3.18964 ^T^	*Ageratina adenophora*	China	OK030885	OK513620	OK513686	OK513649	OK513583	-
*C. yulongense*	CFCC 50818 ^T^	*Vaccinium dunalianum* var. *urophyllum*	China	MH751507	MH777394	MK108986	MK108987	MH793605	MH793604

BRIP—Queensland Plant Pathology Herbarium; CBS—CBS-KNAW Fungal Biodiversity Centre, Utrecht, The Netherlands; CFCC—China Forestry Culture Collection Center; CGMCC—China General Microbiological Culture Collection Center; ICMP—International Collection of Microorganisms from Plants; IMI—International Mycological Institute; MFLUCC—Mae Fah Luang University Culture Collection, Chiang Rai, Thailand; NTUCC—the Department of Plant Pathology and Microbiology, National Taiwan University Culture Collection. ^T^ Ex-type strains. Strains in this study are in bold.

**Table 3:** The best-fit nucleotide substitution model for each dataset, selected by AIC in MrModeltest. 2.2.

Gene	Substitution Model
ITS	SYM+I+G
*act*-exon	HKY
*gapdh*-exon	F81
*β-tubulin*-exon	GTR+G
*chs-1*-exon	K80+G
*cal*-exon	SYM+G
*act*-intron	K80+G
*gapdh*-intron	HKY+G
*β-tubulin*-intron	K80+G
*cal-intron*	SYM+G

## Data Availability

The completed alignments and trees were submitted to TreeBASE submission ID 31014 (http://purl.org/phylo/treebase/phylows/study/TB2:S31014?x-access-code=a44bff36f1453301a23a6c12ba2d815c&format=html accessed on 28 September 2023).
